# Expression of cancer–testis antigens in the immune microenvironment of non‐small cell lung cancer

**DOI:** 10.1002/1878-0261.13474

**Published:** 2023-06-27

**Authors:** Feria Hikmet, Marc Rassy, Max Backman, Loren Méar, Johanna Sofia Margareta Mattsson, Dijana Djureinovic, Johan Botling, Hans Brunnström, Patrick Micke, Cecilia Lindskog

**Affiliations:** ^1^ Department of Immunology, Genetics and Pathology, Rudbeck Laboratory Uppsala University Sweden; ^2^ Department of Medicine (Medical Oncology) Yale University School of Medicine New Haven CT USA; ^3^ Division of Pathology, Department of Clinical Sciences Lund Lund University Sweden

**Keywords:** cancer–testis antigens, immune phenotype, immune‐oncology, non‐small cell lung cancer

## Abstract

The antigenic repertoire of tumors is critical for successful anti‐cancer immune response and the efficacy of immunotherapy. Cancer–testis antigens (CTAs) are targets of humoral and cellular immune reactions. We aimed to characterize CTA expression in non‐small cell lung cancer (NSCLC) in the context of the immune microenvironment. Of 90 CTAs validated by RNA sequencing, eight CTAs (DPEP3, EZHIP, MAGEA4, MAGEB2, MAGEC2, PAGE1, PRAME, and TKTL1) were selected for immunohistochemical profiling in cancer tissues from 328 NSCLC patients. CTA expression was compared with immune cell densities in the tumor environment and with genomic, transcriptomic, and clinical data. Most NSCLC cases (79%) expressed at least one of the analyzed CTAs, and CTA protein expression correlated generally with RNA expression. CTA profiles were associated with immune profiles: high MAGEA4 expression was related to M2 macrophages (CD163) and regulatory T cells (FOXP3), low MAGEA4 was associated with T cells (CD3), and high EZHIP was associated with plasma cell infiltration (adj. *P*‐value < 0.05). None of the CTAs correlated with clinical outcomes. The current study provides a comprehensive evaluation of CTAs and suggests that their association with immune cells may indicate *in situ* immunogenic effects. The findings support the rationale to harness CTAs as targets for immunotherapy.

## Graphical abstract

The study characterizes the expression of cancer–testis antigens (CTAs) in non‐small cell lung cancer in the context of immune cells. We show that CTAs are associated with a specific pattern of immune cell infiltration in the tumor microenvironment. This systematic evaluation represents a framework for identifying CTAs with suggested *in situ* immunogenic effects to serve as potential targets for immunotherapy.

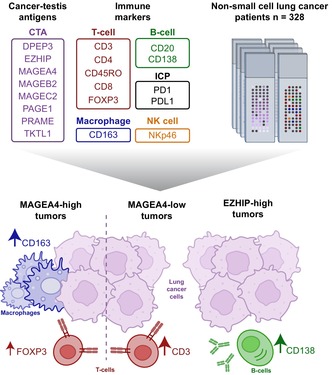

**Abbreviations**
AC: adenocarcinoma
CD: cluster of differentiation
CTA: cancer–testis antigen
DPEP3: dipeptidase 3
EGFR: epidermal growth factor receptor
eTMB: estimated tumor mutational burden
EZHIP: enhancer of zeste inhibitory protein
FDR: false discovery rate
FOXP3: forkhead box protein 3
HPA: Human Protein Atlas
KRAS: Kirsten rat sarcoma viral oncogene
LUAD: lung adenocarcinoma
LUSC: lung squamous cell carcinoma
MAGE: melanoma‐associated antigen
NKp46: natural killer protein 46
NSCLC: non‐small cell lung cancer
NY‐ESO‐1: New York esophageal squamous cell carcinoma‐1
PAGE1: prostate‐associated antigen 1
PD1: programmed death 1
PD‐L1: programmed death ligand 1
PRAME: preferentially expressed in melanoma antigen
SCC: squamous cell carcinoma
STK11: serine/threonine kinase 11
TIMEx: tumor‐immune microenvironment deconvolution method
TKTL1: transketolase ligand 1
TMA: tissue microarray
TP53: tumor protein 53

## 1 Introduction

The introduction of immune checkpoint inhibitors, as single agents or in combination with chemotherapy, provides for the first time a chance of long‐term survival in advanced non‐small cell lung cancer (NSCLC) patients without driver alterations such as epidermal growth factor receptor or ALK [1, 2, 3]. This is, however, only true for a minority of patients [4], and consequently, extensive efforts have been made to identify biomarkers that predict such benefits of immunotherapy and provide clinicians guidance for treatment. Currently approved markers include the expression of programmed death ligand 1 (PD‐L1) on cancer cells and tumor mutational burden [5, 6]. Accumulating evidence also suggests that the local immune environment plays a significant role in the clinical outcome, providing not only independent prognostic information but also advice on which immune cell patterns can be activated by checkpoint inhibitors [7, 8, 9]. Given the relatively low response rates of immunotherapy, alternative treatment strategies are urgently needed to overcome the innate or acquired resistance that develops in several cancers [10, 11]. Taken together, a detailed understanding of cancer immunity will help to optimize the selection of current treatment options and identify additional immune targets.

Cellular and humoral immune responses are intrinsically connected to cancer development, and most cancers can be regarded as immunogenic [12, 13]. Tumor‐associated antigens, recognized by their cellular or humoral immune response, comprise neoantigens that originate due to mutations. Another group of tumor‐associated antigens are endogenous proteins that are not expressed under physiological conditions. Cancer–testis antigens (CTAs) are expressed throughout embryonic development but can be re‐expressed during tumorigenesis. Particularly, melanoma and lung cancer demonstrate high expression of CTAs. Physiological CTA expression in normal tissues is mainly restricted to the testis, and a few are present in placental tissue. The auto‐immunogenic response is suppressed due to testicular cells lacking MHC molecules, and therefore, they do not present antigens to T cells properly. These CTAs are immunogenic as they induce T‐cell‐mediated and humoral immune responses [12, 14, 15, 16, 17]. Based on these unique characteristics, CTAs may serve as potential treatment targets for highly specific immunotherapy and cancer vaccines [12].

In a previous study [14], 90 CTAs were identified by a systematic, comparative RNA sequencing (RNA‐Seq) approach that defined the CTA landscape of NSCLC on the transcriptomic level. However, this molecular analysis to characterize CTAs implies some limitations, as follows: (a) Does the increased CTA gene expression translate to immunogenic protein levels in the cancer cells? (b) Is CTA expression coordinated and related to the histopathologic or genomic features of the cancer cells? (c) Are immunogenic CTAs associated with the immune profiles of the tumor microenvironment, indicating that CTAs induce an *in situ* immune reaction? and (d) Is the expression of CTAs as immune targets associated—in analogy to certain immune cell infiltrates—with a favorable prognosis, which supports the concept of the functional relevance of antigen presentation?

The present study aimed to address these questions through an in‐depth evaluation of the CTA protein landscape in an extensive NSCLC tissue microarray (TMA) patient cohort with detailed clinical and molecular characterization. For protein profiling, we utilized a stringent validation pipeline taking advantage of the Human Protein Atlas (HPA) workflow [18, 19]. The comparison of CTA expression with *in situ* immune cell infiltrates and immune profiles provides information on the impact of CTAs on cancer immunity in NSCLC.

## 2 Materials and methods

### 2.1 Patient material and ethical disclosure

The patient cohort used in the present study has been described previously [14] and is based on consecutive patients with NSCLC that underwent surgical resection at Uppsala University Hospital, Uppsala, Sweden, between the years 2006 and 2010. The median follow‐up time was 10.16 years (interquartile range 9.20–11.52), and the end of the follow‐up period was either patient death or 29 March 2019. The study included TMAs with duplicate 1 mm cores of primary NSCLC tumors from 360 patients, whereby a maximum of 357 patients were immune profiled, and a maximum of 328 were available for CTA profiling. The discrepancy in the number of patients analyzed was due to TMA sample availability. Source data are available upon request. The study was conducted following the Declaration of Helsinki and the Swedish Ethical Review Act approved by the Ethical Review Board in Uppsala (ref for normal tissues from HPA: 2002‐577, 2005‐388, 2007‐159, and 2011‐473; ref for lung cancer tissues: 2012/532). All samples were anonymized for personal identity, and all patients gave their written informed consent.

### 2.2 RNA‐Seq, immunohistochemistry, and mutational analysis

Tissues were obtained from the Clinical Pathology Department, Uppsala University Hospital, Sweden, and collected within the Uppsala Biobank organization and handled following Swedish laws and regulations. For NSCLC tissue, RNA was extracted from fresh frozen tissue corresponding gene expression data for 197 patients (also included in the TMAs) obtained from RNA‐Seq, which has previously been described [14]. The RNA extraction and RNA‐Seq procedure for normal tissues in HPA has also been described previously [19]. For normal tissues, formalin‐fixed, paraffin‐embedded tissue blocks from the pathology archives were selected on the basis of normal histology using a hematoxylin–eosin‐stained tissue section for evaluation. The immunohistochemical protocol has been described previously [19]. The antibodies used for immunohistochemical analysis, dilution factors, and vendor information are available in Table S1. The immune markers were retrieved from previous work [20], and the markers for CTA were from previous CTA candidate targets [14]. Available antibodies within the HPA project were used for screening and filtered on protein‐coding genes according to the Ensembl database with the goal of including 10 proteins in the CTA analysis. Proteins with no available antibody, multitargeting antibodies (i.e., antibodies binding more than one protein), and nonspecific antibodies were used as exclusion criteria by careful examination of the publicly available immunohistochemical images at https://www.proteinatlas.org/. Mutational data from targeted deep‐sequencing have been described previously [21].

### 2.3 Annotation of CTA and immune stainings

Digitized immunohistochemical images were manually annotated by FH (CTA) and quality‐controlled by a second observer. The CTA staining was annotated by scoring the staining highest intensity as negative (0), weak (1), moderate (2), or strong (3). The percentage of positive tumor cells in both TMA cores was also scored by no staining (0), 0–1% (1), 2–10% (2), 11–25% (3), 26–50% (4), 51–75% (5), and > 75% (6). Intensity and quantity scores were multiplied to generate protein scores between 0 and 18. A protein score of 0–2 was considered a low expression, and a protein score of 3–18 was considered a high expression except when otherwise stated. For DPEP3 and PRAME, cytoplasmic staining was scored; for EZHIP, MAGEB2, MAGEC2, and PAGE1, nuclear staining was scored; and for MAGEA4 and TKTL1, nuclear and cytoplasmic staining was scored. For MAGEA4 and TKTL1 staining, with both cytoplasmic and nuclear staining, the maximum protein score value of either was used in the analysis. All immunohistochemical images from the CTA analysis can be retrieved from the BioStudies (https://www.ebi.ac.uk/biostudies) repository (accession S‐BIAD453). Immune annotation was done as described previously [20].

### 2.4 Statistics

The statistical analysis was performed in r (version 4.1.2 ‘Bird Hippie’; RRID: SCR_001905, The R Foundation, Vienna, Austria) and python (version 3.6.9; RRID: SCR_008394, Python Software Foundation, DE, USA) with the modules Pandas 1.1.5, NumPy 1.19.5, and SciPy 1.4.1. A *P*‐value of < 0.05 (with 95% confidence intervals) was defined as significant and used for all statistical analyses if not stated otherwise. For some analyses, the Benjamini–Hochberg procedure for false discovery rate (FDR) was applied to adjust *P*‐values. For clinical outcomes (histological subtype, gender, WHO performance score, age category, smoking status, and tumor stage), data were initially analyzed with Fisher's exact test, followed by FDR, with different protein expression statuses (low vs. high). The treatment variable was not included in the analysis. The r package ‘survminer’ was used for Kaplan–Meier survival models with log‐rank analysis between CTA high and CTA low. This allowed studying the association between high and low CTA protein expressions and overall survival. We further proceeded with a multivariate (age, histology, gender, performance status, smoking, stage, and CTA protein expression) approach using a Cox regression analysis with the previously mentioned clinical criteria. To evaluate the immune cell infiltration score for each CTA protein, we used a Wilcoxon rank‐sum test due to skewed distribution, with confidence intervals and *P*‐values. Immune marker association to either CTA high or CTA low expression was plotted by forest plots with the r packages ‘forestmodel’ and ‘forestplot’. Heatmaps were done with the r package ‘Complexheatmap’ [22], and immune and CTA protein expression scores were scaled and translated into a 0–1 range, followed by an unsupervised Ward‐D clustering and Euclidean distance measurements dictated by the CTA scores. Immune marker scores, mutation status for EGFR, KRAS, STK11, and TP53, and additional clinical data were stacked on top and visualized in the order of CTA clustering. For the heatmap with RNA‐Seq data, FPKM values for CTA and immune genes were log2‐ transformed with added +1 pseudo counts to avoid negative values. For mutational analysis, the estimated tumor mutational burden (eTMB) was calculated by dividing the number of nonsynonymous mutations in a sample by the size (0.47 Mb) of the sequenced genome. To compare each of the 82 genes' mutational status with CTA proteins' expression status (low vs. high), we first performed a Fisher's exact test, followed by FDR. Additionally, the fold change between the eTMB‐averages of the high‐expression and the low‐expression tumors for each protein was calculated. For statistical analysis, we used Levene's test for equality of variance, followed by a *t*‐test for independent samples and FDR. TIMEx data was used to visualize the correlation of CTAs with deconvoluted tumor‐immune microenvironment data from the TCGA. Precomputed *z*‐scores from the TIMEx resource (http://timex.moffitt.org) were used to determine the relation of CTA expression against the different immune signatures developed by TIMEx. The data were generated on TCGA bulk transcriptomics and visualized as a heatmap to indicate correlation value (from −1 to 1) [23]. Only cases that express the CTAs (filtering by RNA‐Seq rsem value ≥ 1) were included and Pearson correlations in adenocarcinoma (AC) and squamous cell carcinoma (SCC) samples (and combined) from the Lung adenocarcinoma (LUAD) and lung squamous cell carcinoma (LUSC) cases of the 2018 PanCancer Atlas (dataset available through cBioportal) were used. The correlation heatmap was generated with the r package ‘corrplot’, and correlations that were significant (*P* adj. < 0.05) were labeled with an asterisk.

## 3 Results

**Fig. 1:**
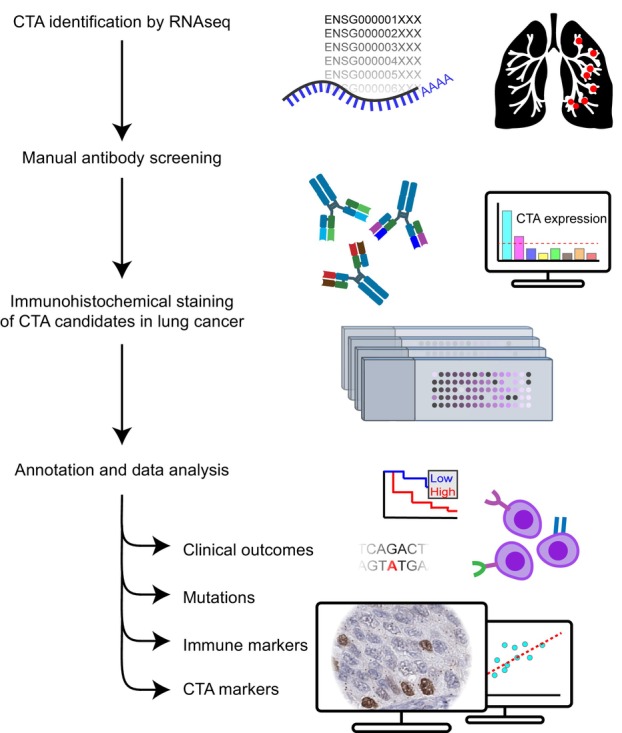
Overview scheme of study. CTAs identified in NSCLC patients and available antibodies for the corresponding CTA proteins were manually assessed by utilizing the HPA portal for inclusion in the study. The selected antibodies were stained on a TMA cohort comprising 328 NSCLC cases, and CTA protein expression and distribution were annotated as described in the Materials and methods section. Clinical parameters used were patient age and gender, cancer stage, lung cancer histological subtype, WHO performance status, and smoking status. Tumor mutations for 82 genes were assessed as well as a calculated combined mutation score. Eight CTAs and 11 immune markers related to B cells and plasma cells, NK cells, T cells, macrophages, and immune checkpoint inhibitor markers were all analyzed by immunohistochemistry.

The study was based on a TMA cohort consisting of 328 NSCLC patients with extensive histopathological and clinical annotations (Table 1). The cohort was utilized for a comprehensive evaluation of the protein expression patterns of selected CTAs, and the study design is presented in Fig. 1. RNA‐Seq and targeted DNA analysis was available for 197 patients and have been described previously [14].

**Table 1:** Clinical characteristics of patients with NSCLC included in CTA and immune protein analysis.

	*n*	%
Total number of patients	328	100.0
Age (median = 67, interquartile range = 62, 74)		
> 70 years	133	40.5
≤ 70 years	195	59.5
Gender		
Female	165	50.3
Male	163	49.7
Histology		
Adenocarcinoma	209	63.7
Adenosquamous carcinoma	5	1.5
Large cell carcinoma	5	1.5
Large cell neuroendocrine carcinoma	9	2.7
Sarcomatoid	2	0.6
Squamous cell carcinoma	98	29.9
Performance status, WHO		
0	205	62.5
1	120	36.6
2	3	0.9
Smoking status		
Smokers	168	51.2
Ex‐smokers	124	37.8
Never‐smokers	36	11.0
Stage (TNM 7)		
IA	141	43.0
IB	68	20.7
IIA	34	10.4
IIB	34	10.4
IIIA	43	13.1
IV	8	2.4
Treatment		
No adjuvant treatment	171	52.1
Adjuvant treatment	129	39.3
Missing data	28	8.6

### 3.1 Selection of CTA candidates

We used a list of 90 CTAs that were identified through a comparative RNA‐Seq‐based approach where genes were defined as CTAs if at least 2% of NSCLC patients and only testis and placenta among normal tissues showed expression above the detection cutoff [14]. Ten genes were removed for being deemed as nonprotein‐coding according to Ensembl version 92.38. Seven genes were removed for which the corresponding protein lacked validated antibodies, and 10 additional genes were excluded because available antibodies (provided by the HPA) showed a high degree of cross‐reactivity. For the remaining 63 genes, we performed a systematic manual assessment of immunohistochemical staining patterns based on the HPA pipeline (version 19; https://v19.proteinatlas.org/). The HPA project generates a map of all human proteins based on antibody‐based proteomics, comprehensively presented in the open‐access database. By validating the CTA expression across 44 different normal tissue types and 20 types of cancer, with a focus on distinct and clear staining in testis and no expression in other normal tissues (except placenta), we selected 37 CTA based on antibody data with the highest specificity. These 37 genes were subjected to re‐titration efforts and further optimized for immunohistochemistry to improve the signal‐to‐noise ratio, after which 19 genes were excluded. Out of the remaining 18 genes, we selected 10 genes with antibodies that showed the clearest and most distinct staining pattern in the testis in the HPA image resource. These 10 genes were further screened on a cohort of 60 NSCLC patients with antibodies targeting their corresponding protein, out of which two proteins were excluded due to all cases being negative (data not shown). The remaining eight proteins showed distinct expression in testis and a subset of NSCLC patients, characterized with stringently validated antibodies. These eight proteins were selected for further analysis, thereby constituting the top candidates for in‐depth profiling. These eight genes were dipeptidase 3 (DPEP3), enhancer of zest homologs inhibitory protein (EZHIP), three melanoma‐associated antigens (MAGEA4, MAGEB2, and MAGEC2), prostate‐associated antigen 1 (PAGE1), preferentially expressed antigen in melanoma (PRAME), and transketolase‐like 1 (TKTL1), corresponding to both highly characterized and poorly characterized proteins with regard to their function (Fig. S1).

### 3.2 Protein expression patterns of CTAs in NSCLC

**Fig. 2:**
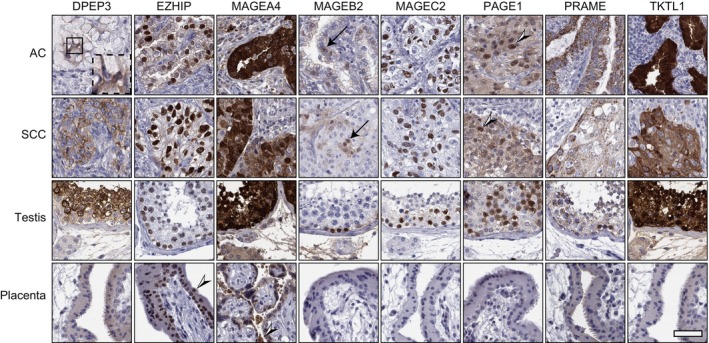
Cancer–testis antigen protein staining in NSCLC and normal tissues. Representative immunohistochemical staining of eight CTAs in AC, SCC, testis, and placenta (counterstained with hematoxylin in blue). All CTAs were positive in testis at variable levels, and EZHIP and MAGEA4 were also positive in placental villi (black and white arrowheads). Clear staining is shown in both AC and SCC cases except DPEP3, which was generally weak in AC (see dashed square for magnified view), and MAGEB2, which was weak in both AC and SCC, but distinctly stained nuclei were present in low fractions (black arrows). PAGE1 protein was present in low fractions in lung cancer (black and white arrowhead). Scale bar = 50 μm.

The eight CTA proteins were stained with automated immunohistochemistry on the NSCLC cohort consisting of 328 patients, and the intensity of the signal was manually annotated. Representative images of staining patterns in NSCLC cases with AC and SCC histology, normal testis, and placenta are shown in Fig. 2. All eight studied CTA markers showed distinct positivity in normal testis, and EZHIP and MAGEA4 were also distinctly stained in the placenta (Fig. 2). The analysis showed that the expression of selected CTAs was variable across the samples, with 79% of cases expressing at least one of the CTAs, and MAGEA4, PRAME, and MAGEC2 constituted the most abundant CTAs, with positivity in 38.1%, 36.8%, and 11.9% of the cases, respectively. Some CTAs were predominantly expressed in AC, such as PRAME, and some preferentially in SCC, such as MAGEA4 (Fig. S2A), indicated through the negative correlation coefficients (Fig. S2B). Furthermore, several other CTAs showed a coordinated expression, that is, they were expressed together with other CTAs. Significant correlations, indicating co‐expression, were observed for EZHIP, PAGE1, MAGEA4, MAGEC2, and TKTL1 when all patients were evaluated (Fig. S2B). When analyzing the CTA expression within separate cancer stages, most CTAs displayed a relatively even distribution. MAGEA4‐positive cases were mainly accumulated between stages 1B to 2B, while for PRAME, there was a relatively clear bias to stage 4 (Fig. S2C). In general, CTA protein expression correlated with RNA expression (Fig. S3). A detailed description of total positive cases per CTA and histological subtype is available in Table S2.

### 3.3 An integrated overview of CTA expression in the clinicopathologic landscape of NSCLC

The NSCLC cohort includes molecular and clinical data linked to each patient. In total, 271 NSCLC cases were evaluable for all eight CTAs, with complete annotation for all 11 immune markers (CD3, CD4, CD8, CD20, CD45RO, CD138, CD163, FOXP3, NKp46, programmed death 1 [PD‐1], and PD‐L1), mutation status, and overall survival. To obtain an integrated overview of CTA expression in the clinicopathological environment of NSCLC, we performed an unsupervised cluster analysis based on CTA expression (Fig. S4). A small group of patients expressed several CTAs, another subset expressed only one or two CTAs, and another one‐third of patients did not express any of the evaluated CTAs. For the patients with available RNA‐Seq data, a comparable pattern was obtained when performing a similar analysis when clustering RNA‐Seq data (Fig. S5). However, in these first overviews, we did not notice a clear association of CTA protein expression with either of the immune cell markers or the given clinical or molecular information. Therefore, we next performed an in‐depth analysis of all available molecular features and clinical parameters.

### 3.4 CTA expression, mutations, clinical parameters, and survival

To evaluate whether CTA expression is connected to specific genomic molecular subtypes of NSCLC, we used Fisher's exact test to test whether CTA expression is associated with mutation data procured from the targeted analysis of 82 lung cancer‐related genes [21] (Table S3). The expression of CTAs was primarily connected to the histological subtypes with a specific mutation pattern. For instance, MAGEA4 was positively associated with TP53 mutations (predominant in SCC) and negatively with KRAS mutations (predominant in AC). When the CTAs' mutation association was analyzed within the AC and SCC subtypes separately, no significant relation was identified after adjustment for multiple testing (Tables S4 and S5). When all mutations were calculated as an eTMB score to assess the average mutational burden between patients, no significance was detected (Table S6). Lastly, clinical parameters (age, gender, smoking status, and stage) were tested for their relation to the expression of a specific CTA. After rigorous adjustment for multiple testing, we identified significant associations only with histology (Table S7). In the next step, we analyzed the CTAs for their prognostic potential. In the Kaplan–Meier survival analysis (Fig. S6) and multivariate Cox regression analysis (Fig. S7 and Table S8), none of the CTA markers showed significant relation to survival.

### 3.5 The association between CTA expression and immune cell infiltration

**Fig. 3:**
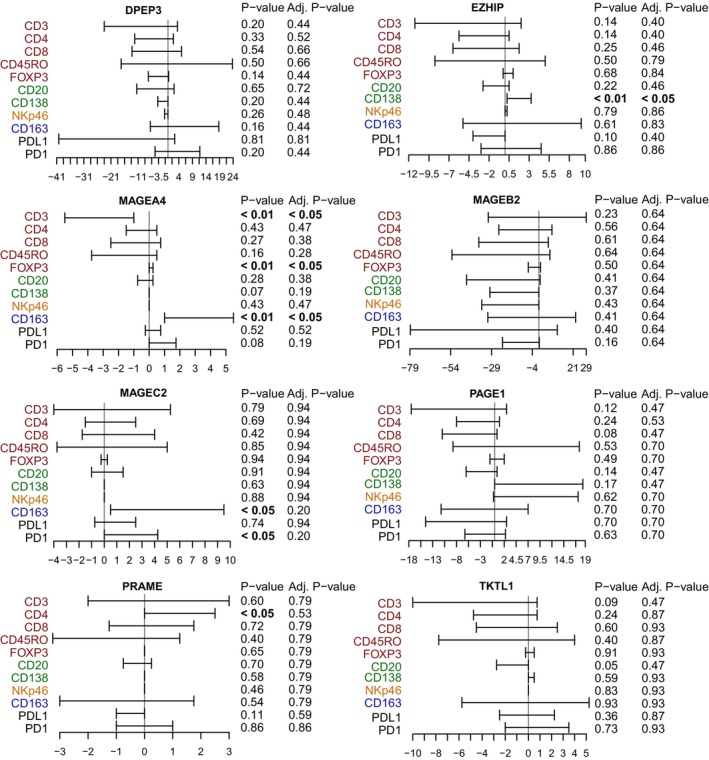
Cancer–testis antigen protein score association to immune cell infiltrates. For each CTA, we performed a Wilcoxon's rank‐sum test against each immune cell marker, T cells (red), B cells (green), NK cells (orange), macrophages (blue), and immune checkpoint inhibitor markers (black). Confidence levels for each immune marker are shown where the median of the difference between CTA high (brackets to the right, above zero) and CTA low (brackets to the left, below zero) is specified. The level of significance by Wilcoxon's rank‐sum test was set at *P* < 0.05 and indicated in bold.

The immune cell microenvironment of the cancer tissue was characterized previously with immunohistochemical markers for CD3 (T lymphocytes), CD4 (T‐helper cells), CD8 (cytotoxic T cells), CD20 (B cells), CD45RO (memory T cells), CD138 (plasma cells), CD163 (M2‐like macrophages), FOXP3 (regulatory T cells), and NKp46 (NK cells) and quantified as the percentage of viable cells in the stroma and tumor compartment separately [20]. In addition, the immune checkpoint protein markers PD‐1 and PD‐L1 were also included in the analysis. To study how the CTA protein expression is associated with immune cell infiltration, we performed a Wilcoxon rank‐sum test for all patients. High EZHIP expression was associated with plasma cell infiltration (CD138) when considering all patients (*P* adj. < 0.05). For MAGEA4 high‐expression cases, the infiltration of CD163‐positive macrophages showed a significant association (*P* adj. < 0.05). High MAGEA4 was also associated with FOXP3 infiltration (*P* adj. < 0.05). Interestingly, a low MAGEA4 signal was highly significant with the pan T‐cell marker CD3 (*P* adj. < 0.05) but not for the other T‐cell markers. High MAGEC2 was associated with CD163‐positive macrophages and PD1‐positive T‐helper cells (*P* < 0.05 for both), but was not significant after adjustment for multiple testing (*P* adj. = 0.20 for both). High PRAME expression was associated with CD4‐positive T‐helper cells (*P* < 0.05), but significance was not reached after adjusting for multiple testing (*P* = 0.53). DPEP3, MAGEB2, and PAGE1 did not show any significant association with any immune marker when considering all cases (Fig. 3). When stratifying the patients based on histology, among AC cases MAGEA4‐high was associated with CD163 (*P* < 0.05) (Fig. S7). Furthermore, low EZHIP was significantly associated with PDL1 expression (*P* < 0.01) and low TKTL1 was linked to the T‐cell markers CD3 and CD45RO (*P* < 0.05). Also, low TKTL1 expression showed a trend toward association with CD4 and CD20 expression but was deemed nonsignificant. In SCC cases, only high EZHIP was linked to CD138 (*P* < 0.05). Note that none of the CTA and immune associations were significant after adjusting for multiple testing when analyzing the data separately by histology. (Fig. S8). To validate our protein profiling results generated by IHC, we applied an in silico approach based on RNA‐Seq deconvolution from the TIMEx web portal (http://timex.moffitt.org) [23]. Preprocessed data from the TIMEx resource were used to determine the relation of CTA gene expression against the different immune signatures. In the complete cohort of AC and SCC cases, most CTAs exhibited no correlation or a subtle negative correlation with different immune signature components (Fig. S9). For PRAME, almost all correlations were significant (*P* adj. < 0.05) with a low inverse correlation. When histological subtypes were analyzed separately (AC or SCC), more cases demonstrated significant CTA‐immune signature correlations. However, the coefficients were in general low and did not confirm our results based on IHC‐based *in situ* CTA and immune cell counts.

## 4 Discussion

The current study presents a detailed and comprehensive characterization of CTAs in their molecular and clinical context and for the first time also in their local tumor microenvironment. The studied CTAs were DPEP3, a membrane‐bound dipeptidase with an unclear function during meiosis [24]; EZHIP (previously named CXorf67) which is involved in the polycomb repressive complex 2 and plays a role in chromatin, histone, and gene silencing biology [25]; MAGEA4, MAGEB2, and MAGEC2, all three belonging to a highly conserved protein family involved in the ubiquitination pathway [26]; PAGE1, which has an unclear function and was first discovered in the androgen‐insensitive prostate cancer cell line LNCaP [27]; PRAME, involved in retinoic acid‐induced cell proliferation arrest, differentiation, and apoptosis [28]; and lastly, the metabolic enzyme TKTL1, which is responsible for the conversion of pentose phosphate molecules in the glycolytic pathway [29]. The selected CTAs were differently expressed in the histological subtypes of lung cancer but were in principle not dependent on the genomic background. Intriguingly, we demonstrated that specific CTAs were associated with the infiltration of different immune cells, including regulatory plasma cells, T cells, and inhibitory macrophage subsets. These findings might indicate an immunogenic impact of CTAs in the local tumor microenvironment, which can potentially be harnessed for therapeutic intervention.

For a long time, CTAs were considered promising targets for cancer therapy. This has been demonstrated for classical CTAs, such as the MAGE family members and the New York esophageal squamous cell carcinoma‐1 (NY‐ESO‐1, gene symbol: CTAG1B), by the detection of autoantibodies in the serum of patients with a variety of cancer types [30, 31]. Likewise, cellular immune reactivity was proven for several CTAs. Indeed, the identification of MAGEA1 in *ex vivo* assays using cytotoxic T cells from melanoma patients [32] was the first evidence that reactive tumor antigens do exist. This finding was the basis for the use of specific cancer antigens as vaccines or to introduce adoptive cell immunotherapies in clinical trials. Unfortunately, although T‐cell responses or antibody induction in cancer patients were observed frequently, the effect of such tumor antigen‐specific strategies on tumor growth was found to be negligible. As a prototypic CTA, the MAGE family member MAGEA3 was used in a large phase III trial (MAGRIT trial), where operated patients with tumors expressing MAGEA3 were vaccinated with the recombinant MAGEA3 protein and the immunostimulant AS15 [33]. These results, together with other vaccination trials, were unfortunately disappointing, lacking significant impact on recurrence‐free or overall survival [34, 35, 36]. It is unclear why in these studies CTA vaccination did not lead to meaningful clinical response despite successful antibody and T‐cell activation. It could be that an optimized screening of patients for the expression of the targeted CTAs would improve response rates [37]. Also, the combination of vaccination with chemotherapy might increase the immunogenicity of the tumor [38]. Alternatively, a preclinical evaluation of an engineered T‐cell receptor therapy against HLA‐A2‐restricted MAGEA4 showed promising results [39]. Currently, the therapy is being used in multiple approaches in clinical intervention trials for patients with different types of tumors [40, 41]. Independent of all these considerations, the most important factor is the choice of CTA.

The accessibility of high‐throughput techniques such as RNA‐Seq makes it attractive to effectively characterize multiple CTAs in minute amounts of tissue [42]. However, a characterization based solely on gene expression data is likely to be insufficient to determine immunogenic protein expression patterns. Proteins constitute the functional counterpart of the genome, and immunohistochemistry has the advantage of showing the exact spatial distribution in the morphologically intact tumor microenvironment. We, therefore, believe an immunohistochemical analysis of cancer tissue is an important requisite to contextualize CTAs. Immunohistochemistry as a method, however, requires thorough antibody validation and calls for caution regarding potential cross‐reactivity and off‐target binding, which may lead to false results. Here, we utilized a rigorous validation pipeline taking advantage of the HPA workflow and put forth major effort in validating the immunohistochemical staining patterns to quantify the best estimate of true protein expression levels. Expectedly, we found that the expression frequency and intensity of the analyzed CTAs are highly variable and correlate only weakly with RNA expression. This aspect should be considered when companion diagnostics for clinical vaccination trials are designed. Interestingly, in the negative MAGE3A MAGRIT trial [33], inclusion was based on the expression on the mRNA level, a strategy that might be insufficient for patient selection and that might partially explain study failure.

In our study, we found that patients with MAGEA4‐positive tumors were harboring TP53 mutations. While this association may be purely correlated with the histological characteristics of cancer subtype [43], it is still interesting to point out that the DNA binding function of TP53 is inhibited by the MAGEA protein family [44]. MAGEA4 also seems to inhibit TP53‐dependent apoptosis. Furthermore, it has been demonstrated that nuclear MAGEA4 expression in the absence of nuclear TP53 expression results in poorer survival of NSCLC patients compared with cytoplasmic MAGEA4 [45]. As we did not analyze the subcellular localization of the CTAs in tumor samples, this could be one factor in why we failed to observe any difference in survival between patients with high or low CTA expression.

We anticipated that if the CTAs were found to be immunogenic, we may detect a specific immune reaction in the tissue. Indeed, we found that some CTAs are associated with the density of immune cells, supporting our assumption. However, these associations were heterogeneous, including M2‐like (CD163+) macrophages and plasma (CD138+) cells. Furthermore, several associations were lost when adjusting for multiple testing. The statistical analysis was also limited by the often very low number of positive cases for some immune and CTA markers, which could lead to hampered statistical associations. Keeping this weakness of our analyses in mind, the identified CTAs that show an immunological signature *in situ* are likely to be immunogenic and could be candidates for focused studies. This is primarily true for MAGEA4, which is associated with decreased numbers of T lymphocytes and increased numbers of M2‐like macrophages. Notably, an association of CTA expression with lower T‐cell counts was not only detected for MAGEA4 but, although not significant, a similar pattern was also observed for DPEP3, EZHIP, PAGE1, and TKTL1. This finding is in contrast to neoantigens, which are formed when nonsynonymous mutations occur. There is evidence that cancers with a high degree of neoantigens expression attract T cells and patients with high neoantigen‐load respond better to immunotherapy [46]. It is yet to be understood if different tumor antigens (neoantigens versus CTAs) induce different cellular immune reactions.

In an attempt to validate the CTA and immune associations, we utilized TIMEx for exploring the CTA gene expression and how it correlated with deconvoluted tumor‐immune microenvironment data. Unfortunately, this analysis did not confirm our findings. Our study intentionally used a direct quantification in the *in situ* environment of lung cancer tissue. The immune cells were counted based on morphology and marker expression in tissue sections. Similarly, the CTAs were quantified at the protein level under microscopic control. We and others have shown that gene and protein expression correlate only moderately [20, 47]. Furthermore, the data from CTAs and immune cells are generated from the same tissue area, while in contrast, the gene expression data cannot be related to the tissue location. These uncertainties for immune cell and CTA gene expression estimations in TIMEx explain that correlations on the protein levels are hidden when using crude RNA‐Seq data.

Our study showed that almost 40% of NSCLC patients showed an expression of MAGEA4, preferentially expressed in cases of SCC histology. Several reports confirmed our findings and showed broad RNA or protein expression of MAGEA4 not only in lung cancer, but also in other cancer types [26, 45, 48, 49, 50, 51]. MAGEA4 is an interesting candidate for targeted immunotherapy, and recently a MAGEA4‐reactive, HLA‐A2‐restricted T‐cell receptor was engineered, showing effectivity and safety in either CD4 or CD8 preclinical assays, suggesting a clinical strategy for an agnostic treatment of MAGE4 positive cancer [52].

We found that many CTAs are predominantly expressed in one of the main histological lung cancer types, indicating histology‐specific CTA immunogenicity. Consequently, we also included histology‐specific analyses. However, due to the small sample sizes, for these patient subgroups and smaller CTA‐positive cases, the statistical power was limited. Therefore, our histology‐related results should be interpreted with caution, with a risk of over‐ and underreporting of associations. In a previous study by Backman and co‐authors, a generally higher T‐cell infiltration was observed in AC compared with SCC. This also supports the notion that immune features are cancer‐type specific [20].

The recently discovered CTA EZHIP demonstrated an association with a higher infiltration of local plasma cells. EZHIP was also identified in our previous CTA discovery study based on a comparative RNA‐Seq approach to a variety of normal and cancerous tissues. EZHIP was previously not regarded as a CTA, and, as with most other CTAs, the EZHIP gene is located on the X chromosome (Xp11.22). EZHIP was biologically mainly characterized by its mutation and overexpression in posterior fossa ependymoma [53]. The function is not clearly understood, but recent studies suggest that EZHIP is connected to the homologous recombination‐mediated DNA repair pathway [54]. Our current study confirmed the expression of EZHIP in NSCLC on the protein level, and the association with plasma cell infiltration (mainly in SCC histology) strengthens the supposition that EZHIP is a CTA candidate with therapeutic potential. The intracellular PRAME has also been studied with a TCR mimic antibody that recognizes PRAME peptides presented in the HLA‐A2 complex [55]. In this study, PRAME was expressed in 37% of all patients, and with a preference to stage 4, consistent with our study. Although our study only included a limited number of advanced‐stage patients, higher PRAME expression in the advanced stage has also been documented in a pan‐cancer meta‐analysis [56].

The present study is the most comprehensive regarding the number of CTAs analyzed and the depth of the characterization based on several molecular, immune, and clinical properties. However, some limitations should be taken into consideration when interpreting its results. We analyzed only eight CTAs, which is less than 10% of the previously identified CTAs in NSCLC [14]. The limitation was mainly based on the availability of reliably validated antibodies for the immunohistochemical analysis. Secondly, we used TMAs comprising only two 1‐mm cores of the whole tissue section from each patient. Thus, we cannot fully capture the entire cancer tissue heterogeneity of CTA expression. This is further emphasized by the single‐cell exploration of LUAD cell lines, showing intratumor and intertumor heterogeneity [57]. Since CTAs are often considered to be stem cell markers [58], they can perhaps only be expressed in a low proportion of cells. On the contrary, *in situ* immunohistochemistry‐based analysis might better reflect the CTA representation in the tumor environment rather than bulk RNA‐based correlation analysis. Here, newer multiplexing technologies might address some of these limitations by visualizing CTA markers indirectly in the context of relevant cancer and immune markers [59]. Finally, the observation that CTA expression is associated with immune cell abundance is intriguing; nonetheless, it does not necessarily prove a causative relation. However, the proportion of cases with positive CTA expression was in general low, hampering statistical power furthermore, and rigorous adjustment might also impede true associative findings.

## 5 Conclusions

We provide a careful characterization of CTA expression in NSCLC and show that CTA expression is common, coordinated, and histology dependent. Some CTAs are connected with the infiltration of specific immune cell subsets, suggesting an *in situ* immune reaction. The described CTAs represent promising immune candidates, and the corresponding stringently validated antibodies may serve as valuable tools in companion diagnostics. Further studies using relevant methods are warranted, not only to validate the results of this study but to also define the immunogenic properties of these CTAs in order to harness them for immunotherapeutic strategies such as vaccination or T‐cell engineering [55].

## Conflict of interest

The authors declare no conflict of interest.

## Author contributions

PM and CL outlined and designed the study with assistance from FH and MR. FH prepared the figures and wrote the first drafts of the manuscript. Revisions were made by all authors but mainly by FH, PM, and CL. FH optimized and annotated the CTA stainings, and MB, JSMM, and DD optimized and annotated the immune stainings. MR generated figures and conducted the statistics in python. MB, FH, and LM generated figures and conducted the statistics in the r statistical environment. JSMM, DD, and HB complied and generated the lung cancer cohort. PM, JB, and HB carried out the histological review of the cases. All authors have read and accepted the final manuscript.

### Peer review

The peer review history for this article is available at https://www.webofscience.com/api/gateway/wos/peer-review/10.1002/1878-0261.13474.

## Supporting information


**Fig. S1.** CTA candidate antibodies screening and selection. The initial 90 CTAs were used as a starting point to screen for available antibodies in the HPA portal. Nonprotein‐coding genes, replaced genes, proteins with no antibody, and multitargeting antibodies were automatically filtered and omitted. The manual assessment was applied to remove nonspecific antibodies and lastly chose the most distinct antibodies concerning staining intensity to simplify annotation. The number on the left indicates the number of proteins left after each filtering process. The proteins highlighted at the bottom were included in the present study. Note that the gene symbol for CXORF67 has been replaced with EZHIP in newer versions of Ensembl.

**Fig. S2.** CTA staining distribution and correlation. (A) Distribution of CTA protein expression (protein score ≥ 1) per histological subtype. Percentages indicate total frequencies regardless of histology. (B) Cross‐correlation plot between the antibodies targeting the eight CTAs. Spearman correlation of CTA protein scores highlights positive (blue) or negative correlation (red) between CTAs. Significant (p < 0.05) correlates are shown with bold and black text, while nonsignificant are in gray. (C) The number and percentage distribution of stages represented in the lung cancer cohort (upper multicolored bar). Stacked percentage bar charts with positive and negative cases in each bar, grouped by stage for each CTA. Y‐axis represents percentages and the blue‐filled bars represent positive cases defined as protein score ≥ 2 (lower eight plots).

**Fig. S3.** Correlation between CTA protein score and RNA‐Seq levels. Spearman's rank correlations between RNA‐Seq and CTA protein scores. The RNA‐Seq data for each patient of the corresponding boxplots show the distribution of RNA‐Seq for the protein correlation divided by low CTA (red box) and high CTA (green box) protein expression. Circles indicate raw data points and diamonds indicate outliers. The respective p‐values are indicated for each correlate.

**Fig. S4.** Unsupervised hierarchical cluster analysis of the immune marker and CTA protein expression profile in NSCLC patients. Immune annotation scores and CTA protein scores were linearly transformed for each marker and plotted as heatmaps to visualize the global immune and CTA phenotypes in NSCLC patients. The immune data (red matrix) were stacked on top to visualize the immune distribution dictated by the unsupervised hierarchical cluster analysis of the CTA protein score matrix (blue matrix). The upper rows (green matrix) show stage, performance status, histological subtype, gender, age, and mutation status (yellow matrix) for four commonly analyzed genes in lung cancer—EGFR, KRAS, STK11, and TP53. Each column indicates one patient and the total number of patients used is defined at the bottom.

**Fig. S5.** Unsupervised hierarchical cluster analysis of immune and CTA gene expression profiles in NSCLC patients. FPKM gene expression levels of the immune and CTA markers were log2‐transformed and plotted as heatmaps to visualize the CTA‐to‐immune relationship in NSCLC patients. The immune gene expression data (red matrix) were stacked on top to visualize the immune distribution dictated by the unsupervised hierarchical cluster analysis of the CTA genes (blue matrix). The upper rows (green matrix) show stage, performance status, histological subtype, gender, age, and mutation status (yellow matrix) for four commonly analyzed genes in lung cancer—EGFR, KRAS, STK11, and TP53. Each column indicates one patient and the total number of patients used is defined at the bottom.

**Fig. S6.** Kaplan–Meier survival analysis in NSCLC cases with high and low CTA protein scores. Nonparametric log‐rank Kaplan–Meier 5‐year survival analysis was performed to compare survival between high (red) and low (turquoise) CTA protein expression scores. The ‘Number at risk’ table shows the number of alive or noncensored patients at a specific time point for the CTA high or low group. The upper and lower 95% confidence interval is shown as dotted lines.

**Fig. S7.** Multivariate Cox regression analysis. The association of CTAs with multiple variables was analyzed in a multivariate Cox regression analysis. Hazard ratios are presented as log values with 95% confidence intervals.

**Fig. S8.** CTA protein score association to immune cell infiltrates by histology. Wilcoxon's rank‐sum test for each CTA was tested against the immune cell markers, T cells (red), B cells (green), NK cells (orange), macrophages (blue), and immune checkpoint inhibitor markers (black). Confidence levels for each immune marker are shown where the median of the difference between CTA high (brackets to the right, above zero) and CTA low (brackets to the left, below zero) is specified. For adenocarcinoma, 207 patients were analyzed, and for squamous cell carcinoma, 97 patients were analyzed. The level of significance by Wilcoxon's rank‐sum test was set at p < 0.05 and indicated in bold.

**Fig. S9.** CTA gene expression correlation against deconvoluted tumor‐immune microenvironment data. The heatmap shows the relation of CTA gene expression against different immune signatures, all retrieved from the TIMEx web portal. Pearson's correlation coefficient matrix was calculated and indicated as values 1 to −1 (blue to red). A red asterisk indicates a significant correlation (p. adj <0.05). Only cases that express the specific CTA were included in the analysis. The number of patients analyzed per CTA in total was as follows: EZHIP; 117, MAGEB2; 121, DPEP3: 229, TKTL1: 265, MAGEC2:162, PRAME: 446, MAGEA4: 141, and PAGE1: 60.

**Table S1.** Antibody list for immune and CTA profiling by immunohistochemistry. All antibodies used for tissue profiling and research resource identifier (RRID), catalog information, and dilution are listed.
**Table S2.** Number of positive cases per CTA and histological subtype. The number of positive cases (protein score ≥ 1) by histological subtype per CTA is listed. Please note: individual cases express several CTAs. Number of annotated cases from TMA for each CTA, n = 310.
**Table S3.** CTA protein expression and mutational status in all NSCLC patients. For each CTA, the protein expression status (protein score ≥ 2 = high vs. protein score 0–1 = low) was tested against mutational status in all NSCLC patients by Fisher's exact test. Significant p‐values and FDR‐adjusted p‐values cases are highlighted in green.
**Table S4.** CTA protein expression and mutational status in adenocarcinoma patients. For each CTA, the protein expression status (high vs. low) was tested against mutational status in adenocarcinoma patients by Fisher's exact test. Significant p‐values and FDR‐adjusted p‐values cases are highlighted in green.
**Table S5.** CTA protein expression and mutational status in squamous cell carcinoma patients. For each CTA, the protein expression status (high vs. low) was tested against mutational status in squamous cell carcinoma patients by Fisher's exact test. Significant p‐values and FDR‐adjusted p‐values cases are highlighted in green.
**Table S6.** CTA protein expression and estimated tumor mutational burden. An estimated tumor mutational burden was calculated, and the average mutational burden between patients with high or low CTA expression was evaluated with a *t*‐test. Significant p‐values and FDR‐adjusted p‐values cases are highlighted in green. NA = not applicable.
**Table S7.** CTA protein expression and clinical data. For each CTA, the protein expression status (high vs. low) was tested against clinical criteria (histological subtype, gender, WHO performance score, age category, smoking status, and tumor stage) by Fisher's exact test. Significant p‐values and FDR‐adjusted p‐values cases are highlighted in green.
**Table S8.** Cox regression analysis. Detailed information for Cox regression analysis in Fig S7. N = 308 patients for all analyses. Significant p‐values are highlighted in green.

## Data Availability

All data needed to evaluate the conclusions in the paper are present in the paper and/or the [Link], [Link], [Link], [Link], [Link], [Link], [Link], [Link], [Link], [Link]. Additional data related to this paper are available from the corresponding author (cecilia.lindskog@igp.uu.se) upon reasonable request.
