# Supplementary material for: Expression of cancer–testis antigens in the immune microenvironment of non‐small cell lung cancer
Source: Mol Oncol. 2023 Jun 27;17(12):2603–17. doi: 10.1002/1878-0261.13474 (PMC10701773; doi:10.1002/1878-0261.13474)
Supplement: Supplementary file 1 — Fig. S1. CTA candidate antibodies screening and selection. The initial 90 CTAs were used as a starting point to screen for available antibodies in the HPA portal. Nonprotein‐coding genes, replaced genes, proteins with no antibody, and multitargeting antibodies were automatically filtered and omitted. The manual assessment was applied to remove nonspecific antibodies and lastly chose the most distinct antibodies concerning staining intensity to simplify annotation. The number on the left indicates the number of proteins left after each filtering process. The proteins highlighted at the bottom were included in the present study. Note that the gene symbol for CXORF67 has been replaced with EZHIP in newer versions of Ensembl. [file MOL2-17-2603-s005.pdf]

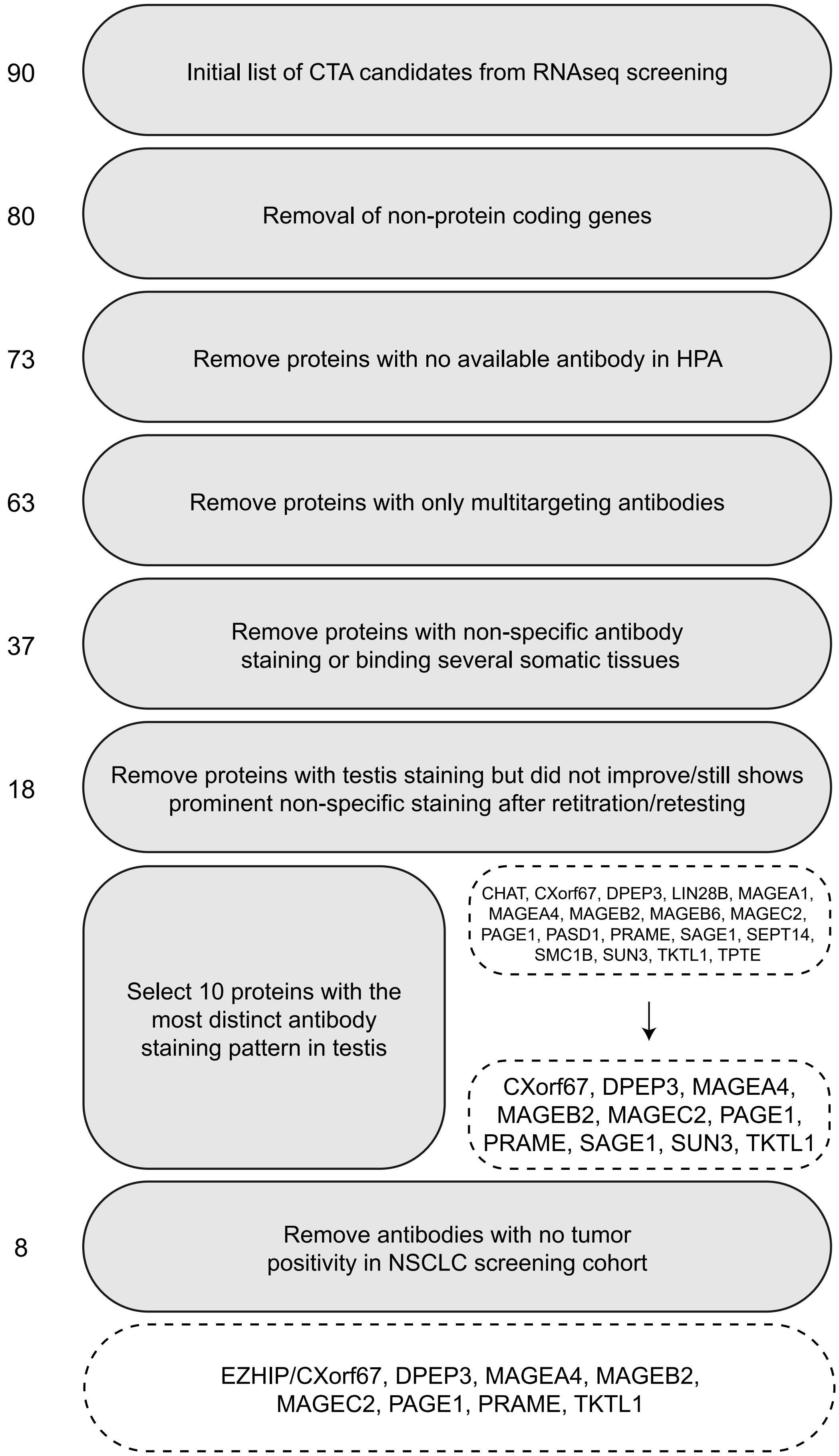
90
Initial list of CTA candidates from RNAseq screening
80
Removal of non-protein coding genes
73
Remove proteins with no available antibody in HPA
63
Remove proteins with only multitargeting antibodies
37
Remove proteins with non-specific antibody staining or binding several somatic tissues
18
Remove proteins with testis staining but did not improve/still shows prominent non-specific staining after retitration/retesting
Select 10 proteins with the most distinct antibody staining pattern in testis
CHAT, CXorf67, DPEP3, LIN28B, MAGEA1, MAGEA4, MAGEB2, MAGEB6, MAGEC2, PAGE1, PASD1, PRAME, SAGE1, SEPT14, SMC1B, SUN3, TKTL1, TPTE
CXorf67, DPEP3, MAGEA4, MAGEB2, MAGEC2, PAGE1, PRAME, SAGE1, SUN3, TKTL1
8
Remove antibodies with no tumor positivity in NSCLC screening cohort
EZHIP/CXorf67, DPEP3, MAGEA4, MAGEB2, MAGEC2, PAGE1, PRAME, TKTL1
