# Supplementary figures and images for: Expression of cancer–testis antigens in the immune microenvironment of non‐small cell lung cancer
Source: Mol Oncol. 2023 Jun 27;17(12):2603–17. doi: 10.1002/1878-0261.13474 (PMC10701773; doi:10.1002/1878-0261.13474)

A

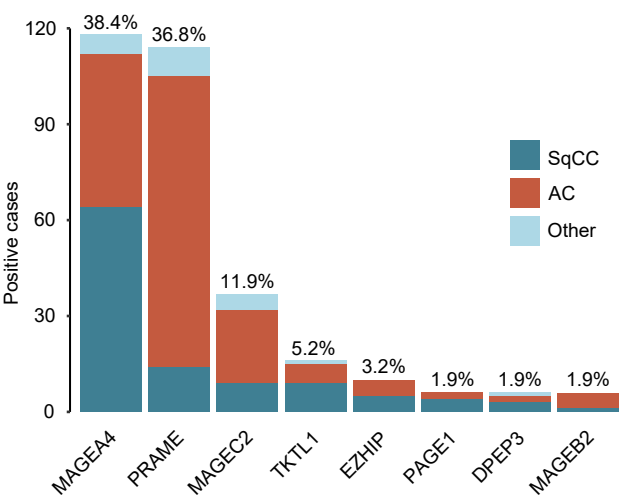

B

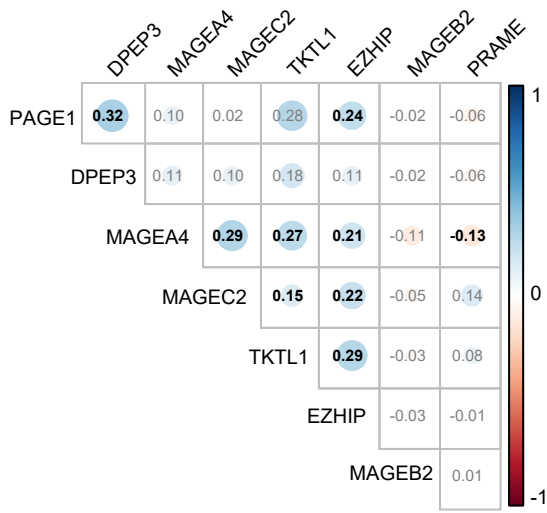

C

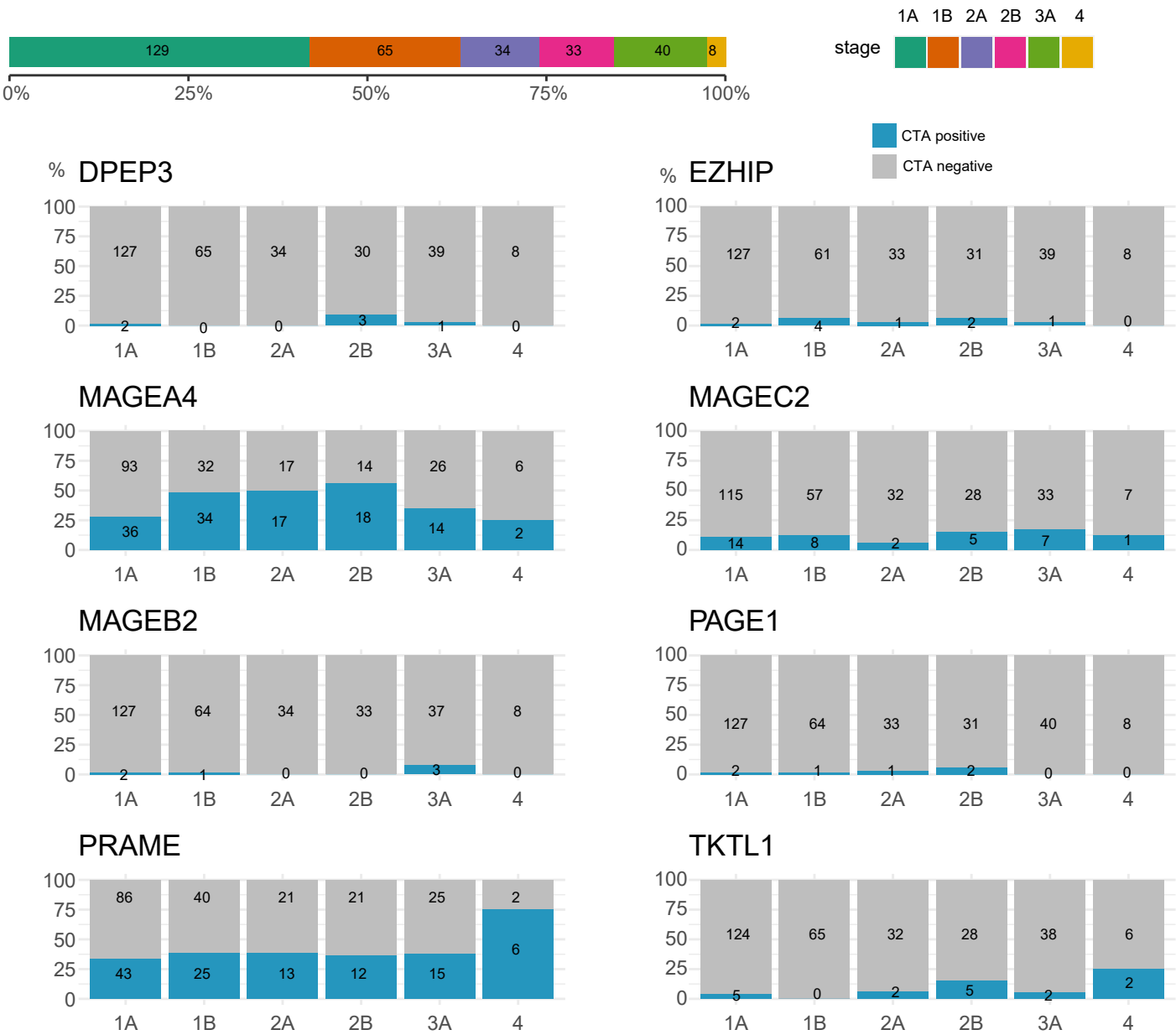

Supplement: Supplementary file 2 — Fig. S2. CTA staining distribution and correlation. (A) Distribution of CTA protein expression (protein score ≥ 1) per histological subtype. Percentages indicate total frequencies regardless of histology. (B) Cross‐correlation plot between the antibodies targeting the eight CTAs. Spearman correlation of CTA protein scores highlights positive (blue) or negative correlation (red) between CTAs. Significant (p < 0.05) correlates are shown with bold and black text, while nonsignificant are in gray. (C) The number and percentage distribution of stages represented in the lung cancer cohort (upper multicolored bar). Stacked percentage bar charts with positive and negative cases in each bar, grouped by stage for each CTA. Y‐axis represents percentages and the blue‐filled bars represent positive cases defined as protein score ≥ 2 (lower eight plots). [file MOL2-17-2603-s009.pdf]

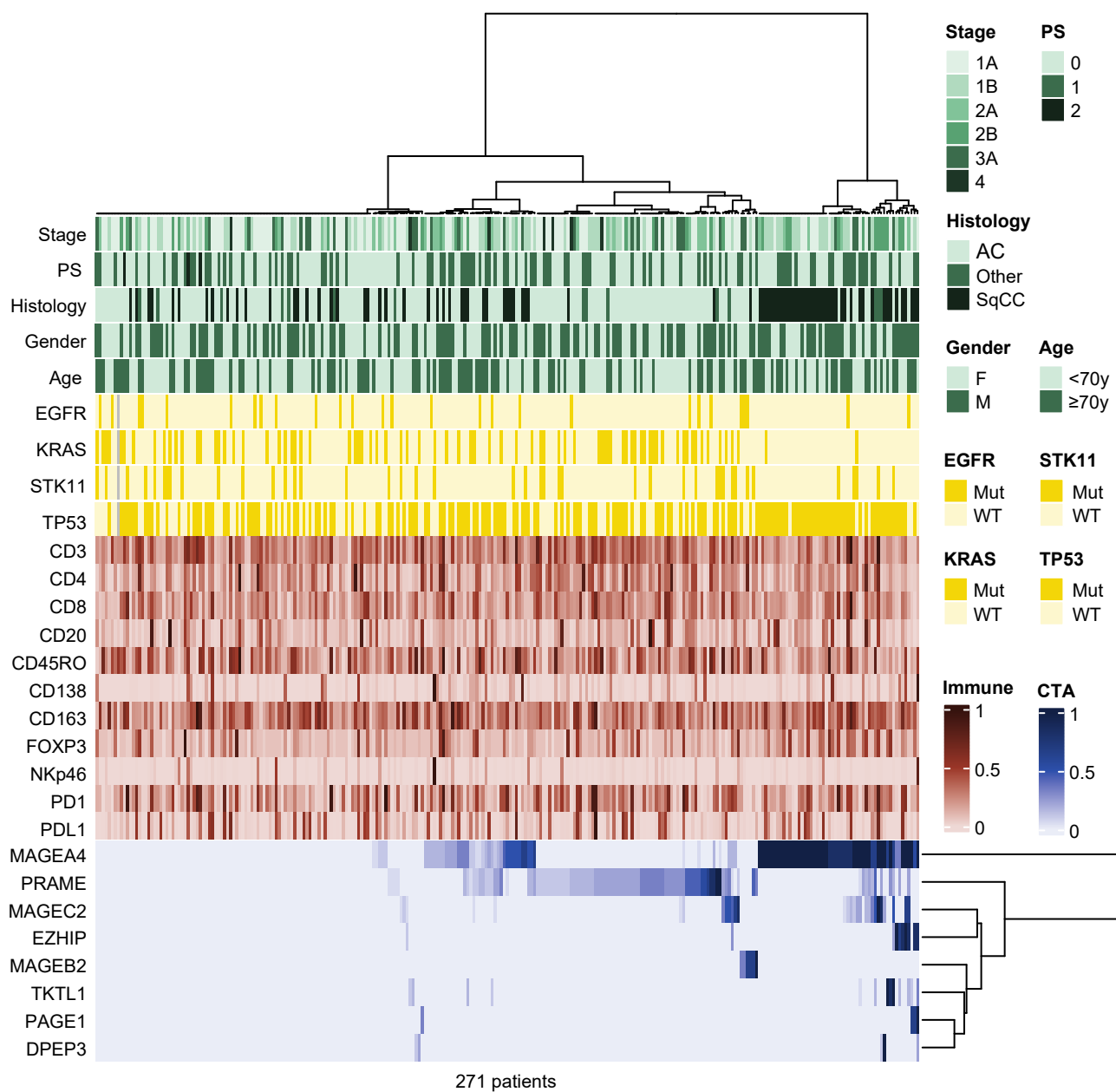

Supplement: Supplementary file 4 — Fig. S4. Unsupervised hierarchical cluster analysis of the immune marker and CTA protein expression profile in NSCLC patients. Immune annotation scores and CTA protein scores were linearly transformed for each marker and plotted as heatmaps to visualize the global immune and CTA phenotypes in NSCLC patients. The immune data (red matrix) were stacked on top to visualize the immune distribution dictated by the unsupervised hierarchical cluster analysis of the CTA protein score matrix (blue matrix). The upper rows (green matrix) show stage, performance status, histological subtype, gender, age, and mutation status (yellow matrix) for four commonly analyzed genes in lung cancer—EGFR, KRAS, STK11, and TP53. Each column indicates one patient and the total number of patients used is defined at the bottom. [file MOL2-17-2603-s008.pdf]

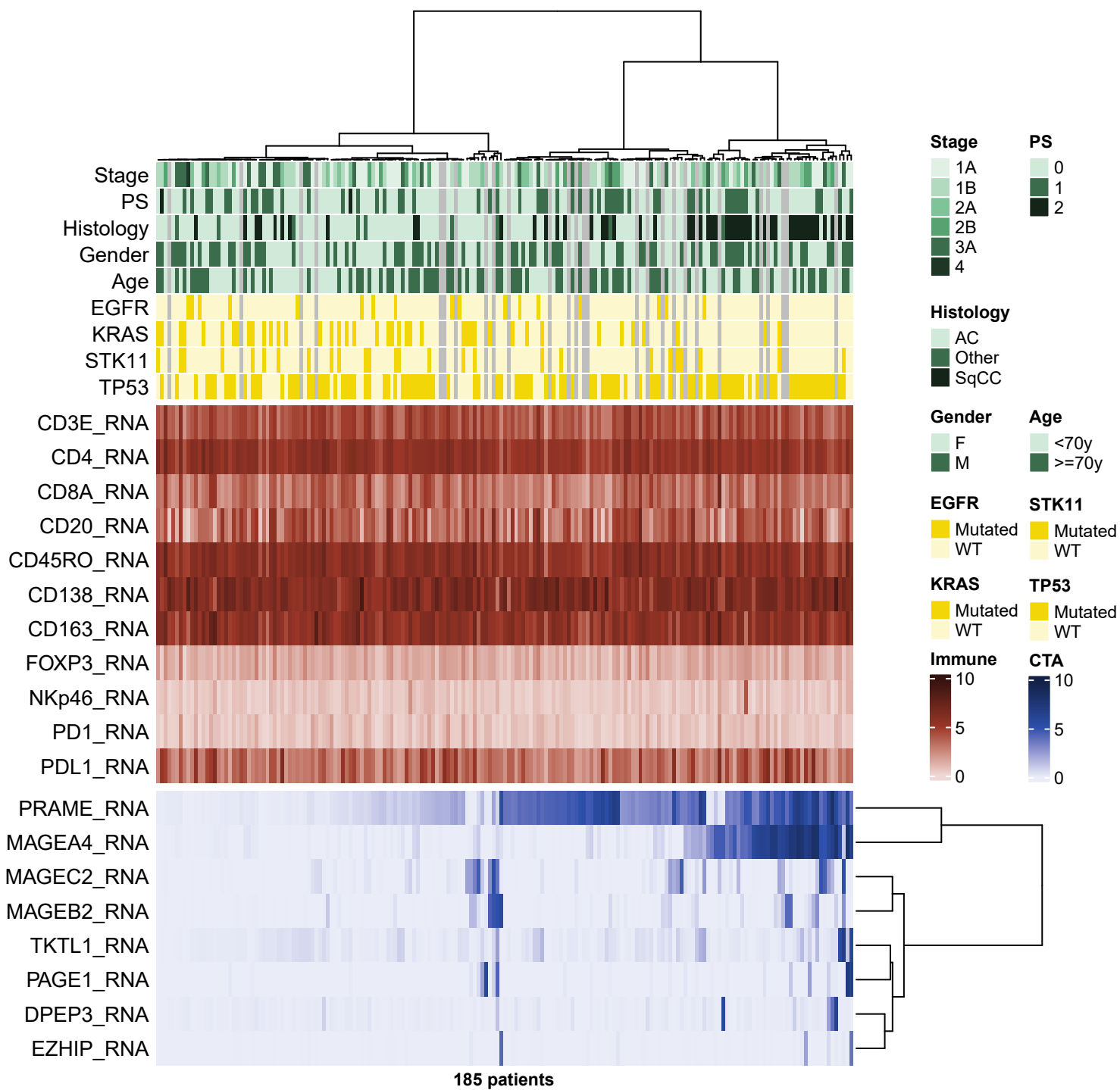

Supplement: Supplementary file 5 — Fig. S5. Unsupervised hierarchical cluster analysis of immune and CTA gene expression profiles in NSCLC patients. FPKM gene expression levels of the immune and CTA markers were log2‐transformed and plotted as heatmaps to visualize the CTA‐to‐immune relationship in NSCLC patients. The immune gene expression data (red matrix) were stacked on top to visualize the immune distribution dictated by the unsupervised hierarchical cluster analysis of the CTA genes (blue matrix). The upper rows (green matrix) show stage, performance status, histological subtype, gender, age, and mutation status (yellow matrix) for four commonly analyzed genes in lung cancer—EGFR, KRAS, STK11, and TP53. Each column indicates one patient and the total number of patients used is defined at the bottom. [file MOL2-17-2603-s003.pdf]

DPEP3

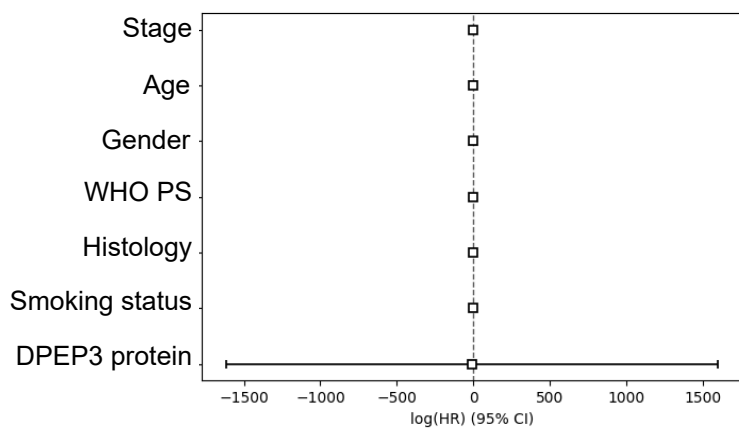

EZHIP

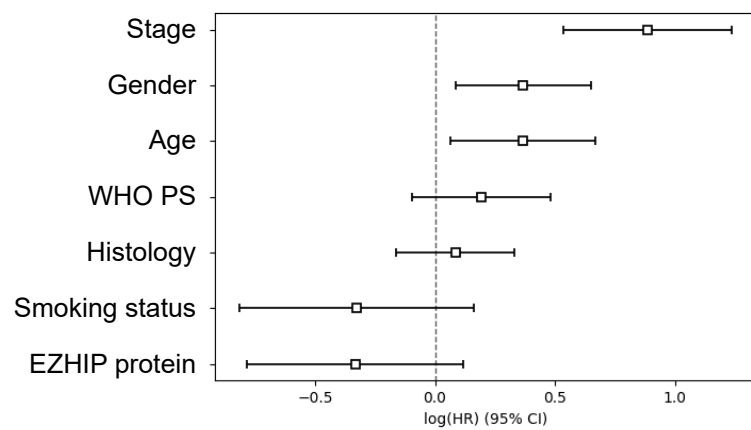

MAGEA4

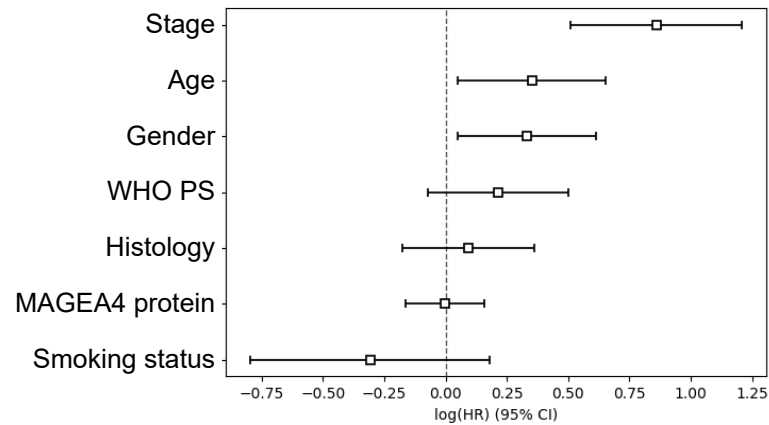

MAGEB2

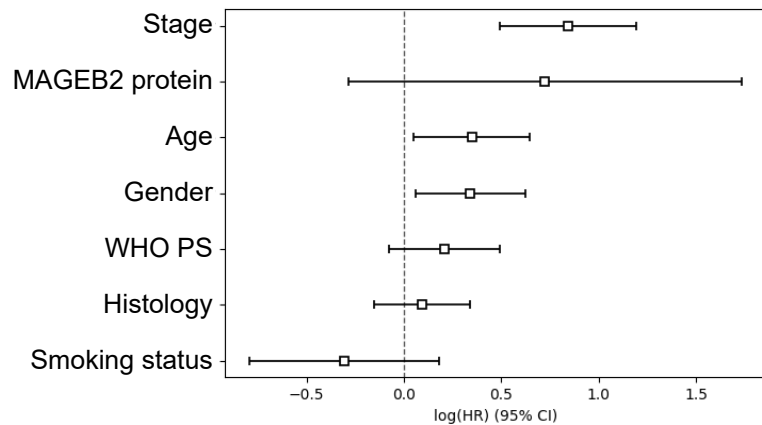

MAGEC2

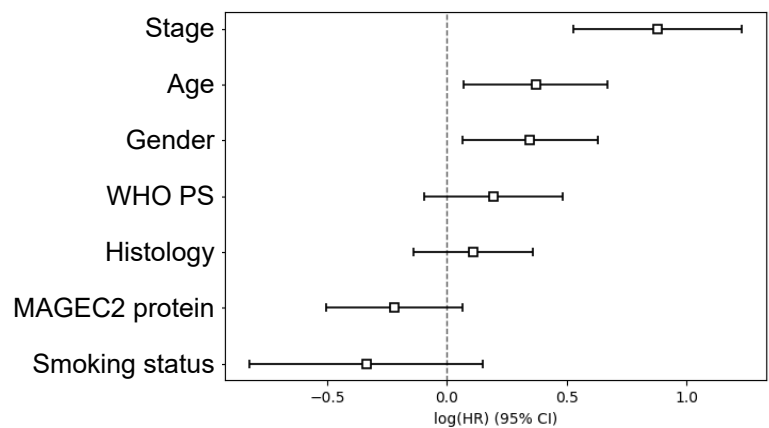

PAGE1

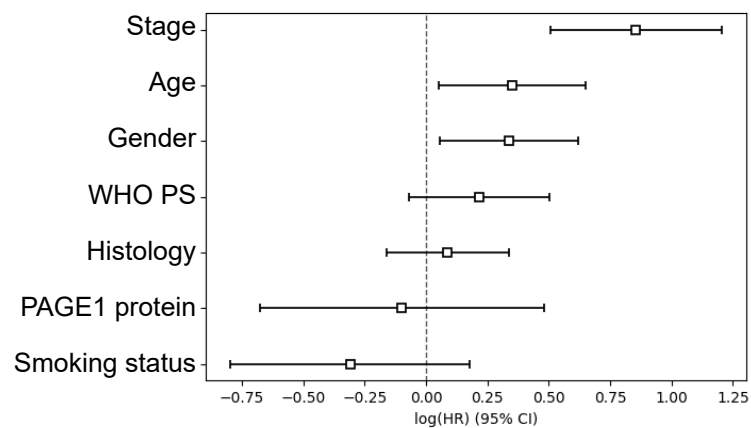

PRAME

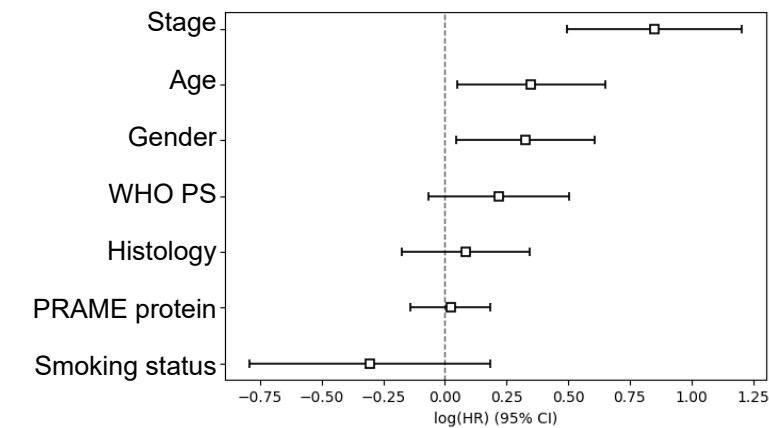

TKTL1

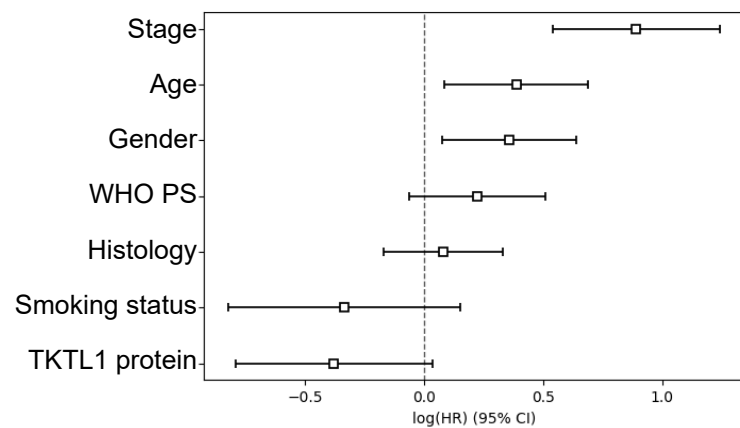

Supplement: Supplementary file 7 — Fig. S7. Multivariate Cox regression analysis. The association of CTAs with multiple variables was analyzed in a multivariate Cox regression analysis. Hazard ratios are presented as log values with 95% confidence intervals. [file MOL2-17-2603-s002.pdf]

Only adenocarcinomas (n=207 patients)

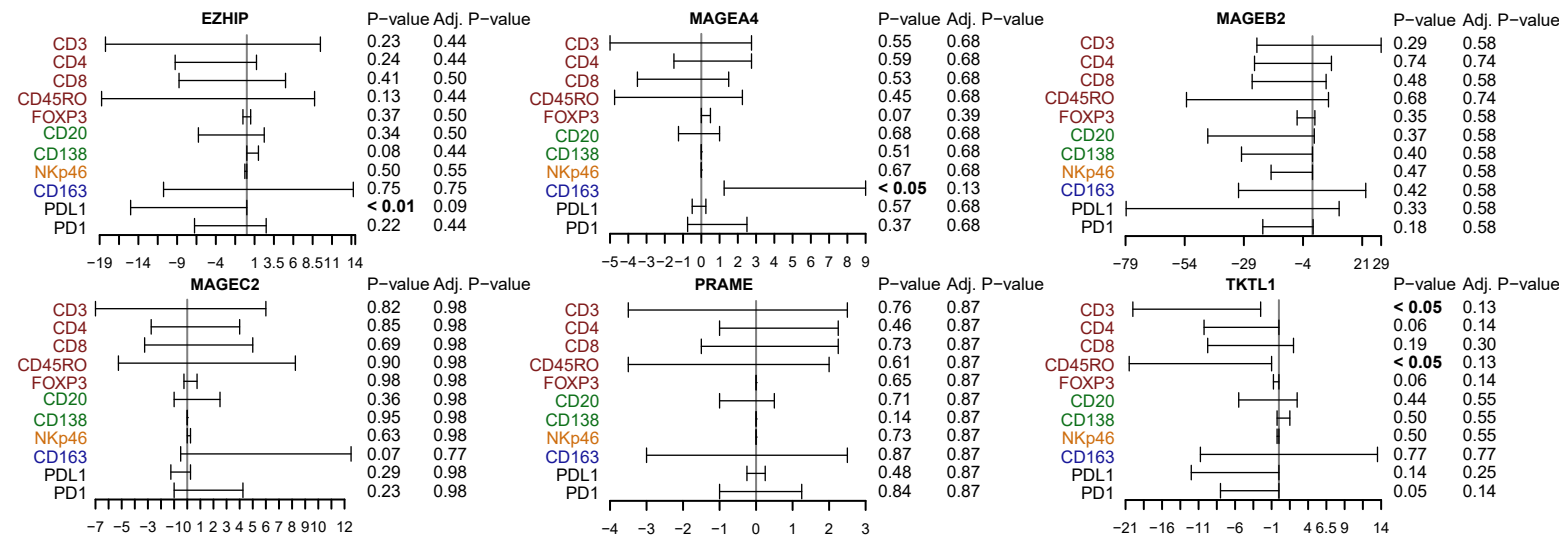

Only squamous cell carcinomas (n=97 patients)

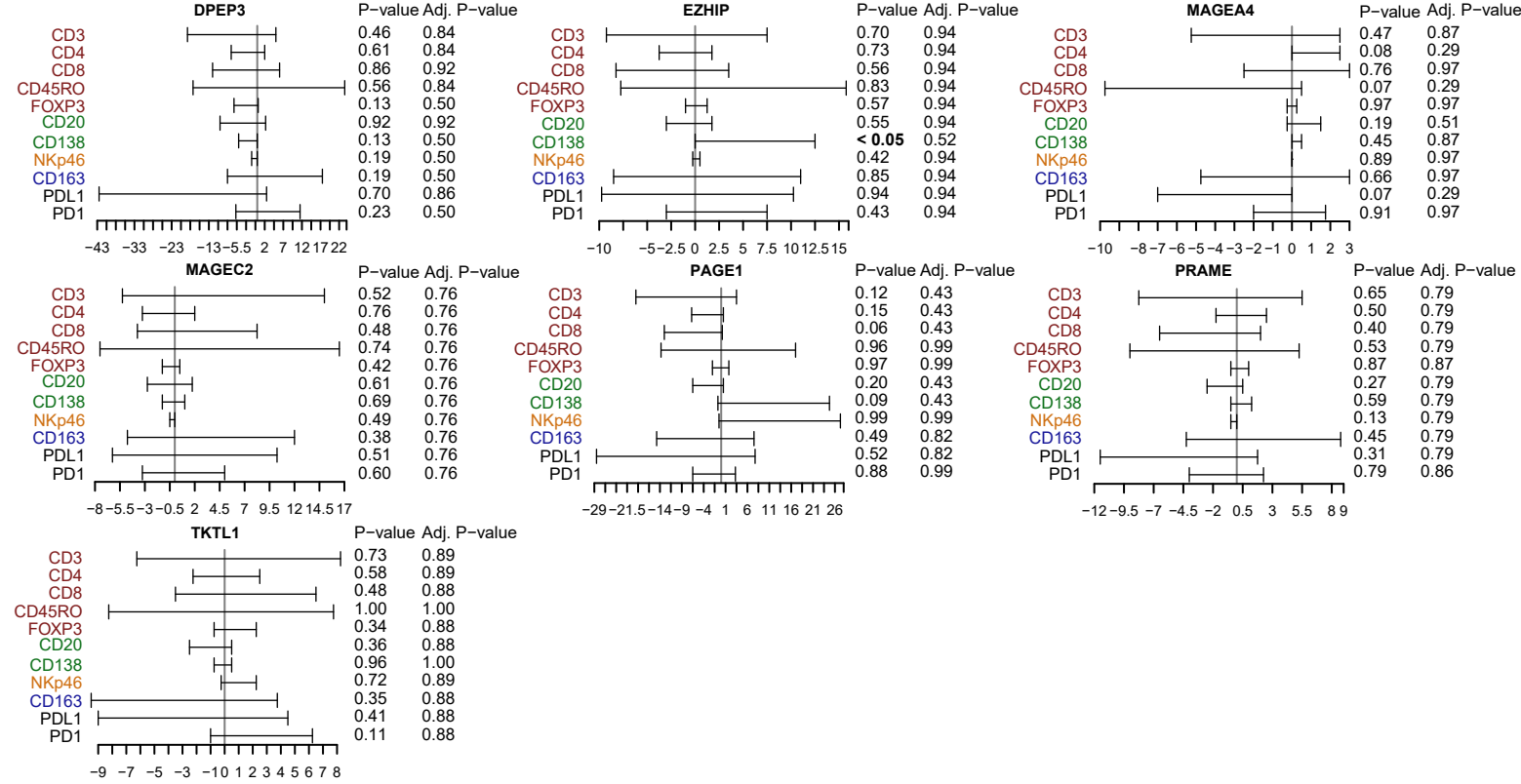

Supplement: Supplementary file 8 — Fig. S8. CTA protein score association to immune cell infiltrates by histology. Wilcoxon's rank‐sum test for each CTA was tested against the immune cell markers, T cells (red), B cells (green), NK cells (orange), macrophages (blue), and immune checkpoint inhibitor markers (black). Confidence levels for each immune marker are shown where the median of the difference between CTA high (brackets to the right, above zero) and CTA low (brackets to the left, below zero) is specified. For adenocarcinoma, 207 patients were analyzed, and for squamous cell carcinoma, 97 patients were analyzed. The level of significance by Wilcoxon's rank‐sum test was set at p < 0.05 and indicated in bold. [file MOL2-17-2603-s001.pdf]

Pearson's *r* \* = p. adj <0.05

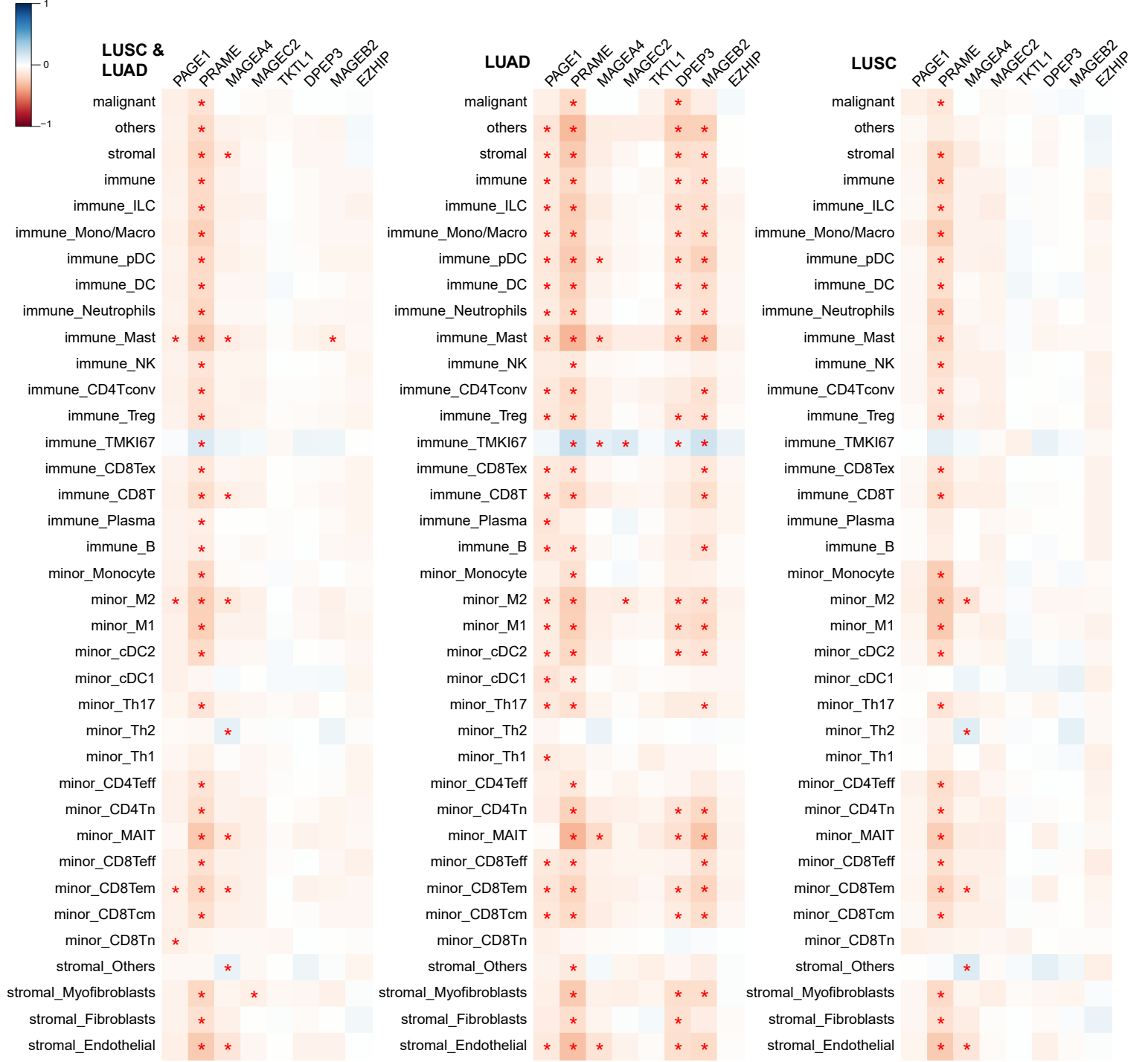

Supplement: Supplementary file 9 — Fig. S9. CTA gene expression correlation against deconvoluted tumor‐immune microenvironment data. The heatmap shows the relation of CTA gene expression against different immune signatures, all retrieved from the TIMEx web portal. Pearson's correlation coefficient matrix was calculated and indicated as values 1 to −1 (blue to red). A red asterisk indicates a significant correlation (p. adj <0.05). Only cases that express the specific CTA were included in the analysis. The number of patients analyzed per CTA in total was as follows: EZHIP; 117, MAGEB2; 121, DPEP3: 229, TKTL1: 265, MAGEC2:162, PRAME: 446, MAGEA4: 141, and PAGE1: 60. [file MOL2-17-2603-s006.pdf]
