# Supplementary material for: Expression of cancer–testis antigens in the immune microenvironment of non‐small cell lung cancer
Source: Mol Oncol. 2023 Jun 27;17(12):2603–17. doi: 10.1002/1878-0261.13474 (PMC10701773; doi:10.1002/1878-0261.13474)
Supplement: Supplementary file 3 — Fig. S3. Correlation between CTA protein score and RNA‐Seq levels. Spearman's rank correlations between RNA‐Seq and CTA protein scores. The RNA‐Seq data for each patient of the corresponding boxplots show the distribution of RNA‐Seq for the protein correlation divided by low CTA (red box) and high CTA (green box) protein expression. Circles indicate raw data points and diamonds indicate outliers. The respective p‐values are indicated for each correlate. [file MOL2-17-2603-s004.pdf]

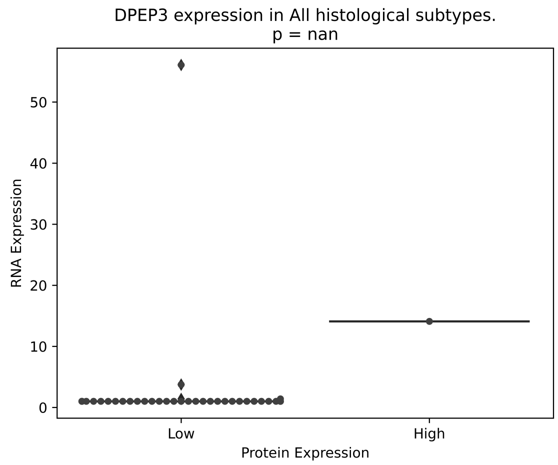
DPEP3 expression in All histological subtypes.
p = nan
RNA Expression
0
10
20
30
40
50
Low
High
Protein Expression

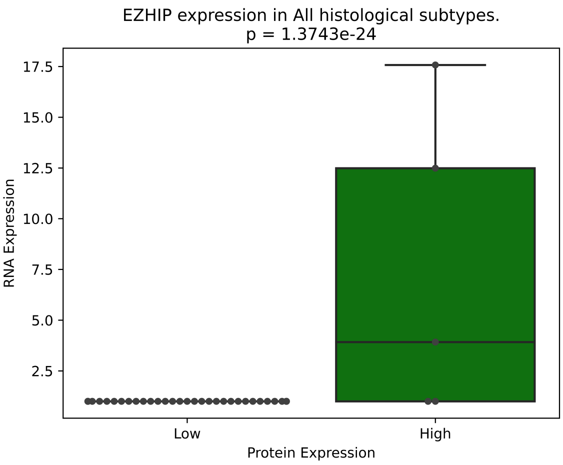
EZHIP expression in All histological subtypes.
p = 1.3743e-24
RNA Expression
2.5
5.0
7.5
10.0
12.5
15.0
17.5
Low
High
Protein Expression

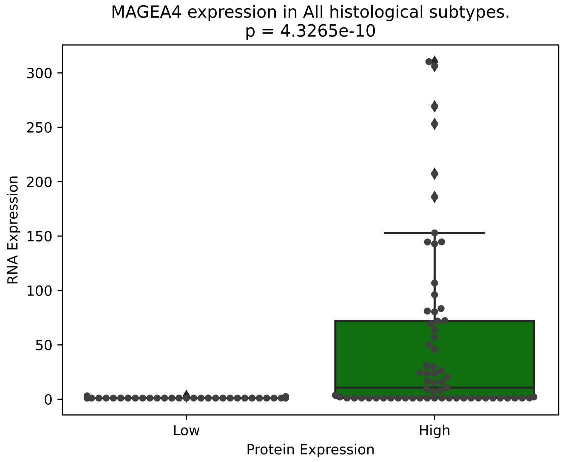
MAGEA4 expression in All histological subtypes.
p = 4.3265e-10
RNA Expression
0
50
100
150
200
250
300
Low
High
Protein Expression

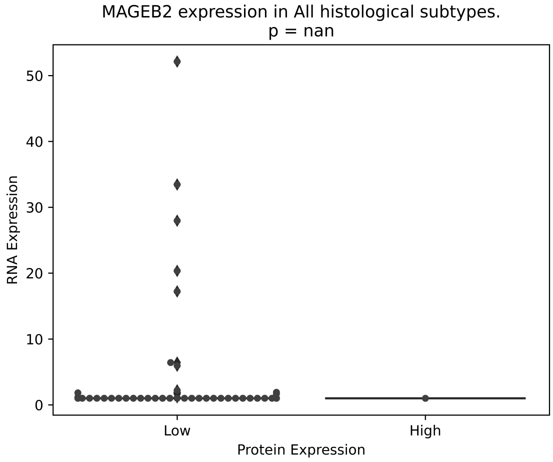
MAGEB2 expression in All histological subtypes.
p = nan
RNA Expression
0
10
20
30
40
50
Low
High
Protein Expression

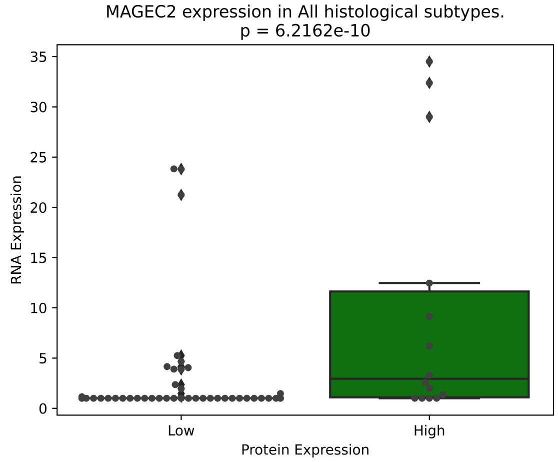
MAGEC2 expression in All histological subtypes.
p = 6.2162e-10
RNA Expression
0
5
10
15
20
25
30
35
Low
High
Protein Expression

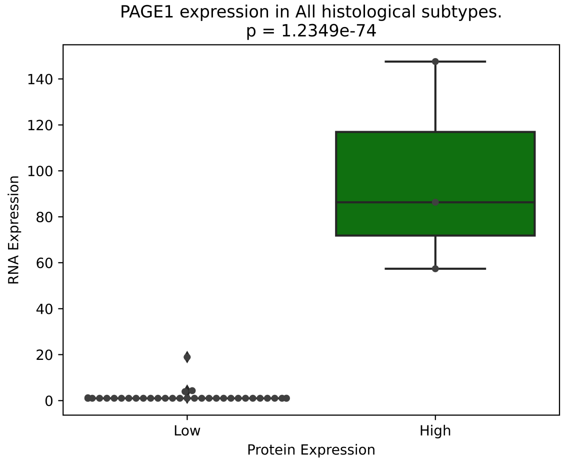
PAGE1 expression in All histological subtypes.
p = 1.2349e-74
RNA Expression
0
20
40
60
80
100
120
140
Low
High
Protein Expression

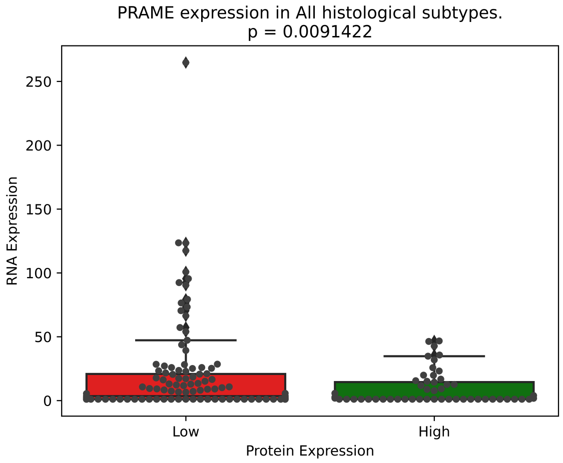
PRAME expression in All histological subtypes.
p = 0.0091422
RNA Expression
0
50
100
150
200
250
Low
High
Protein Expression

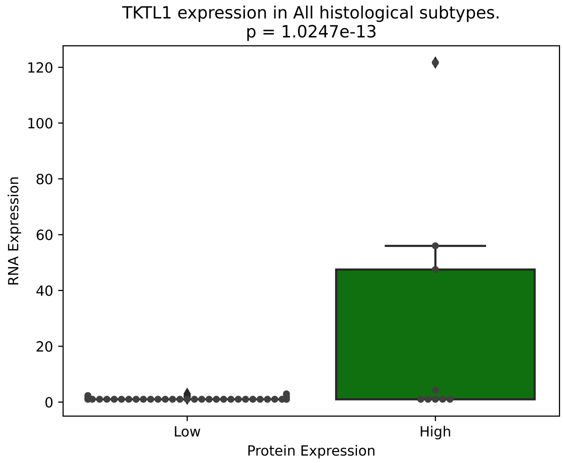
TKTL1 expression in All histological subtypes.
p = 1.0247e-13
RNA Expression
0
20
40
60
80
100
120
Low
High
Protein Expression
