# Supplementary material for: Expression of cancer–testis antigens in the immune microenvironment of non‐small cell lung cancer
Source: Mol Oncol. 2023 Jun 27;17(12):2603–17. doi: 10.1002/1878-0261.13474 (PMC10701773; doi:10.1002/1878-0261.13474)
Supplement: Supplementary file 6 — Fig. S6. Kaplan–Meier survival analysis in NSCLC cases with high and low CTA protein scores. Nonparametric log‐rank Kaplan–Meier 5‐year survival analysis was performed to compare survival between high (red) and low (turquoise) CTA protein expression scores. The ‘Number at risk’ table shows the number of alive or noncensored patients at a specific time point for the CTA high or low group. The upper and lower 95% confidence interval is shown as dotted lines. [file MOL2-17-2603-s007.pdf]

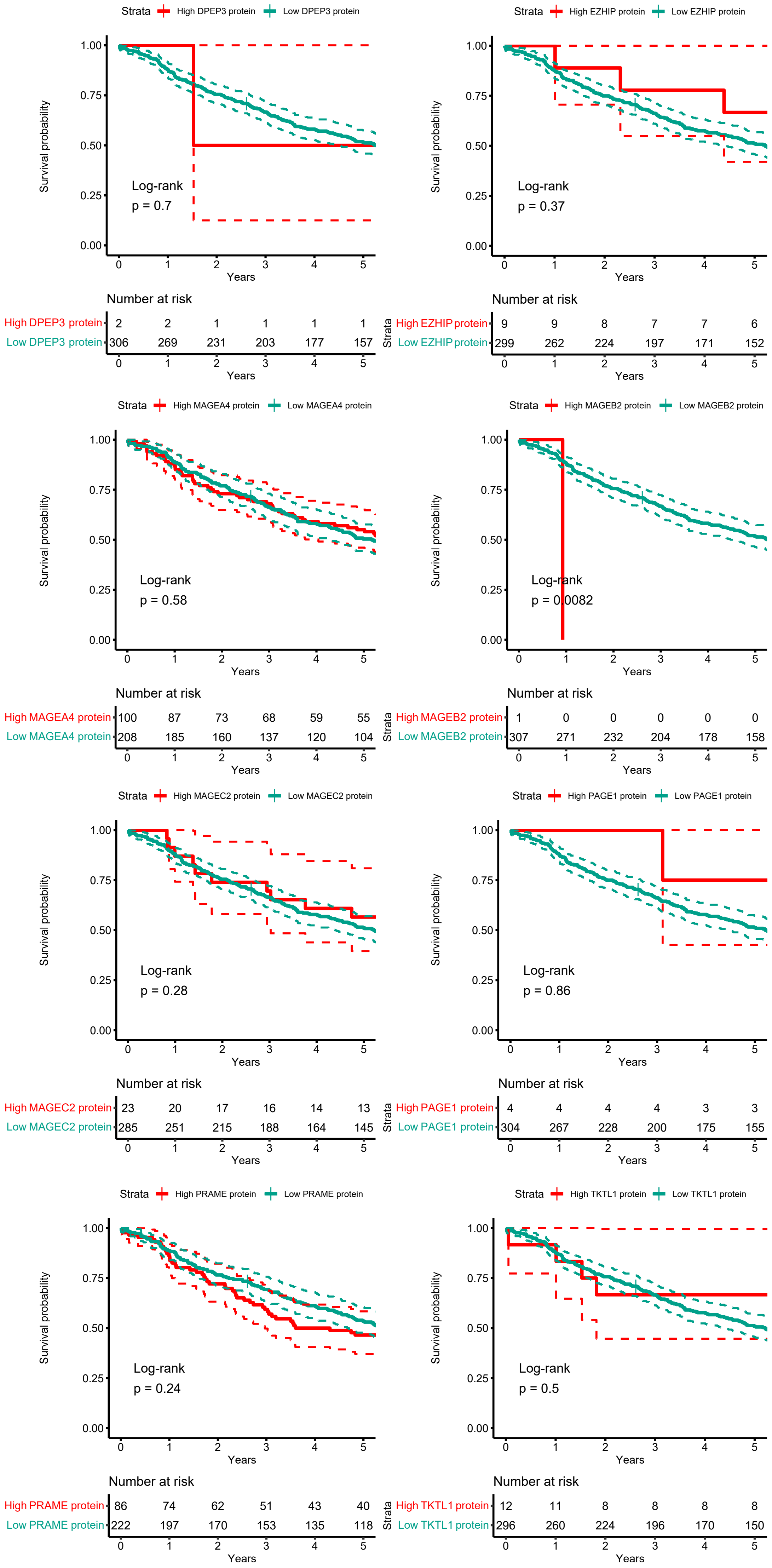
Strata High DPEP3 protein Low DPEP3 protein
Survival probability
Log-rank p = 0.7
Years
Number at risk
High DPEP3 protein 2 2 1 1 1 1
Low DPEP3 protein 306 269 231 203 177 157
Strata High EZHIP protein Low EZHIP protein
Log-rank p = 0.37
High EZHIP protein 9 9 8 7 7 6
Low EZHIP protein 299 262 224 197 171 152
Strata High MAGEA4 protein Low MAGEA4 protein
Log-rank p = 0.58
High MAGEA4 protein 100 87 73 68 59 55
Low MAGEA4 protein 208 185 160 137 120 104
Strata High MAGEB2 protein Low MAGEB2 protein
Log-rank p = 0.0082
High MAGEB2 protein 1 0 0 0 0 0
Low MAGEB2 protein 307 271 232 204 178 158
Strata High MAGEC2 protein Low MAGEC2 protein
Log-rank p = 0.28
High MAGEC2 protein 23 20 17 16 14 13
Low MAGEC2 protein 285 251 215 188 164 145
Strata High PAGE1 protein Low PAGE1 protein
Log-rank p = 0.86
High PAGE1 protein 4 4 4 4 3 3
Low PAGE1 protein 304 267 228 200 175 155
Strata High PRAME protein Low PRAME protein
Log-rank p = 0.24
High PRAME protein 86 74 62 51 43 40
Low PRAME protein 222 197 170 153 135 118
Strata High TKTL1 protein Low TKTL1 protein
Log-rank p = 0.5
High TKTL1 protein 12 11 8 8 8 8
Low TKTL1 protein 296 260 224 196 170 150
